# Chromatin accessibility prediction via a hybrid deep convolutional neural network

**DOI:** 10.1093/bioinformatics/btx679

**Published:** 2017-10-23

**Authors:** Qiao Liu, Fei Xia, Qijin Yin, Rui Jiang

**Affiliations:** 1 MOE Key Laboratory of Bioinformatics; Bioinformatics Division and Center for Synthetic & Systems Biology, TNLIST; Department of Automation, Tsinghua University, Beijing, China; 2 Department of Electrical Engineering, Stanford University, Stanford, CA, USA

## Abstract

**Motivation:**

A majority of known genetic variants associated with human-inherited diseases lie in non-coding regions that lack adequate interpretation, making it indispensable to systematically discover functional sites at the whole genome level and precisely decipher their implications in a comprehensive manner. Although computational approaches have been complementing high-throughput biological experiments towards the annotation of the human genome, it still remains a big challenge to accurately annotate regulatory elements in the context of a specific cell type via automatic learning of the DNA sequence code from large-scale sequencing data. Indeed, the development of an accurate and interpretable model to learn the DNA sequence signature and further enable the identification of causative genetic variants has become essential in both genomic and genetic studies.

**Results:**

We proposed Deopen, a hybrid framework mainly based on a deep convolutional neural network, to automatically learn the regulatory code of DNA sequences and predict chromatin accessibility. In a series of comparison with existing methods, we show the superior performance of our model in not only the classification of accessible regions against background sequences sampled at random, but also the regression of DNase-seq signals. Besides, we further visualize the convolutional kernels and show the match of identified sequence signatures and known motifs. We finally demonstrate the sensitivity of our model in finding causative noncoding variants in the analysis of a breast cancer dataset. We expect to see wide applications of Deopen with either public or in-house chromatin accessibility data in the annotation of the human genome and the identification of non-coding variants associated with diseases.

**Availability and implementation:**

Deopen is freely available at https://github.com/kimmo1019/Deopen.

**Supplementary information:**

Supplementary data are available at *Bioinformatics* online.

## 1 Introduction

Over the past decade, genome-wide association studies (GWAS) have provided genome-wide profiles about the genetic basis of complex traits and common diseases (Manolio, 2010; Stranger *et al.*, 2011). However, building accurate models to interpret functions and properties of the identified genetic variants is still a challenging task due to the complicated mechanism of eukaryotic gene expression, especially the incomplete understanding of non-coding DNA (Ward and Kellis, 2012a). Systematic annotations of functional elements could help us understand regulatory mechanisms underlying genetic signals that are statistically associated with a disease (Paul *et al.*, 2014). It has been argued that the occurrence of a genetic variant may result in the disruption of its hosting regulatory element, and hence cause the development of a disease (Alexander *et al.*, 2010). Consequently, the current inability to precisely predict the implication of regulatory elements directly impedes the progress towards precise medicine and personal medical treatment.

Putative accessible regions in the genome often work together with transcription factors (TFs), RNA polymerases and other cellular machines to regulate gene expression (Kellis *et al.*, 2014). This understanding, together with the fact that disease-associated genetic variants tend to enrich in accessible regions, makes the deciphering of DNA sequence signature such as chromatin accessibility essential for studying functional implications of genetic variants. The identification of chromatin accessibility can be traced back to a class of methods based on the comparison the sequence conservation across different species (Lee *et al.*, 2011). However, the fact that accessibility could not be determined by sequence conservation alone impairs accuracy of these methods and restricts their applications. Recently, the development of high-throughput sequencing technologies, such as DNase-seq, MNase-seq and ATAC-seq, has enabled the accumulation of a vast amount of chromatin profiles across different cell lines. Given chromatin profiles as training data, machine learning models could effectively predict the chromatin accessibility, transcription factor binding sites (TFBS), histone markers and DNA methylation from genome sequences (Ghandi *et al.*, 2014; Kircher *et al.*, 2014; Lee *et al.*, 2011, 2015; Liu *et al.*, 2017; Ward and Kellis, 2012b; Whitaker *et al.*, 2015). A powerful predictive method could help us annotate effects of genetic variants with single-nucleotide sensitivity, especially for rare variants whose functional implications are still unknown.

Over the past 5 years, artificial neural networks with stacked layers have achieved unprecedented performance in many fields including but not limited to computer vision (Sun *et al.*, 2014) and natural language processing (Collobert *et al*., 2011). Previous applications of deep learning models have achieved great success in predicting protein-binding sites, histone markers and DNA accessibility (Alipanahi *et al.*, 2015; Kelley *et al.*, 2016; Quang and Xie, 2016; Zhou and Troyanskaya, 2015). It inspires us that building such predictive models could help us dissect regulatory code of accessible genome which could improve the interpretation of functional genomic sites.

In this article, we introduce Deopen (**De**ep **open**ness prediction network), a computational framework that applies a hybrid deep convolutional neural network (CNN) to learn regulatory sequence code and predict chromatin accessibility at the whole genome level. Through comprehensive experiments, we demonstrate that Deopen not only achieves state-of-the-art performance in the chromatin accessibility classification problem, but also successfully recovers continuous degree of chromatin accessibility for an input sequence, thereby filling the gap of predicting DNA accessibility signals in continuous values. To make Deopen more understandable, we propose a strategy to visualize motifs discovered by our model and successfully find their counterparts in the JASPAR database. To demonstrate applications of Deopen, we focus on a GWAS dataset of breast cancer and show the ability of our method to explain functional implications of putative disease-associated single nucleotide polymorphisms (SNPs). We finally summarize that Deopen, as an effective predictive model for learning DNA regulatory code, could shed light on the understanding of gene regulation mechanisms and the deciphering of genetic basis of human complex diseases.

## 2 Materials and methods

### 2.1 Data preparation and preprocessing

In order to learn DNA sequence codes that determine accessible (open) and inaccessible (closed) chromatins, we randomly select 50 DNase-seq experiments (see detail in Supplementary Table.xls) from the ENCODE Project (Dunham, 2012) across 50 different human cell lines as suggested in Basset (Kelley *et al.*, 2016). Considering that an experiment may have one or more replicates, we merge all sequencing reads for different replicates that correspond to an experiment and apply Hotspot (John *et al.*, 2011), a peak calling tool, to extract putative open regions with FDR equals 0.01 from the merged data. Cell-type specificity can be observed as the cover rate of open regions for different cell types range from 4.6 to 19.8%, which intuitively inspires us that cell-type specific model should be established. To further preprocess the data, we extract 1000 bp from the midpoint of each open region with hg19 reference genome for each DNase I hypersensitive site (DHS), forming the positive training samples. Negative training samples are randomly selected from background sequences in hg19 reference genome. In classification and regression experiments, the original dataset of each cell line is randomly down-sampled to 100k sites due to the limited scalability of gkm-SVM (Lee *et al.*, 2015). We then use 90% of the resulting data for training and the rest 10% for test. All the assessment and analysis are performed on the test set.

### 2.2 Design of Deopen

**Fig. 1.**
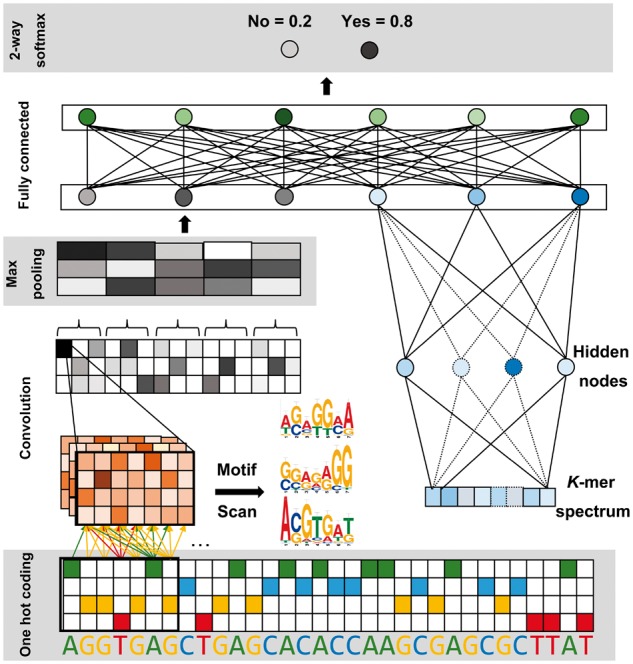
The schematic of Deopen. On the left, a convolutional neural network is applied to automatically learn the DNA sequence feature. On the right, a typical three-layer feed forward neural network is constructed to learn high-level representation of *k*-mer features. We concatenate outputs of the above two networks to form the hybrid fully connected layer. The output layer estimates open probability using a softmax classifier and update through the backpropagation strategy

Deopen has a hybrid architecture which consists of a deep convolutional neural network (CNN) and a typical three-layer feed forward network (Fig. 1). The deep CNN is organized in a sequential layer-by-layer structure where convolution layers and pooling layers play a key role in extracting input features at different spatial scales. The three-layer feed forward network consists of neurons that are connected to every neuron in the next layer. We concatenate the outputs of the above two networks to form a hybrid feature vector as the input of a fully connected layer. The output layer of Deopen consists of a softmax classifier which could estimate the chromatin open probability (see detailed parameters in Supplementary Fig. S5). Deopen not only considers spatial interactions and orientations between sequence patterns but also takes high-level representation of *k*-mers into account. In the implementation of Deopen, we calculate the one-hot matrix and *k*-mer features of input DNA sequences in advance, then we reshape all the inputs into one matrix which is convenient for model training. Besides, we randomly drop half of units in hybrid fully connected layer using dropout (Hinton *et al.*, 2012). More importantly, we introduce a strategy to better initialize weights of convolutional kernels. Briefly, we first generate five models with identical architecture and initialize each model with different weights at random. Then we train each model for three epochs respectively to obtain a rough evaluation of these models. Finally, we select the model with the highest performance in the internal validation and use this model as the starting point to conduct the training procedure. 

The convolution operation in CNN could be denoted as

$$\text{Convolution}{\left(X\right)}_{ik}=\text{Relu}(\sum_{m=0}^{M-1}\sum_{n=0}^{N-1}w_{mn}^{k}x_{i+m,n}),$$

where X is the input matrix, *M* the size of the sliding window, *N* the number of input channels, $W^{k}={\left(w_{mn}^{k}\right)}_{M\times N}$ the weight matrix of the *k*th convolution kernel with size M×N. For the first convolution layer, *N* is equal to 4. For other layers, *N* is equal to the number of convolutional kernels of the previous layer. Relu represents rectified linear unit, which sets negative values to zeros, as

$$\text{Relu}(x)=\{\begin{array}{cc} x & \text{if}\;x\geq 0\\ 0 & \text{otherwise} \end{array}.$$

The maximum value in a window of adjacent positions is calculated for each kernel, for the purpose of reducing the output size and integrate features in a higher level. The pooling operation is denoted as

$$\text{pooling}{\left(X\right)}_{ik}=\max(x_{iM,k},x_{iM+1,k},\ldots ,x_{iM+M-1,k}).$$

The fully connected layers integrate high-level features of DNA sequences and transform the features into a fixed dimension space. The output layer estimates the accessible probability using the softmax regression. The solution to the Deopen classification model can then be regarded as an optimization problem with the objective function

$$\text{arg}\;\text{max}\;\sum_{i=0}^{n}\text{CE}(Y_{i},{\hat{Y}}_{i}),$$

where $Y_{i}$ and ${\hat{Y}}_{i}$ denote the true label and the predicted value of the *i*th sample, respectively, $CE(Y_{i},{\hat{Y}}_{i})=-Y_{i}\;\text{log}\;{\hat{Y}}_{i}-(1-Y_{i})\;\text{log}\;(1-{\hat{Y}}_{i})$_,_ the cross entropy of $Y_{i}$ and ${\hat{Y}}_{i}$, and *n* the number of training samples. We use Adam (Kingma and Ba, 2014) as the optimizer for updating kernel weights.

For the Deopen regression model, instead of binary label, we define openness as below

$$\text{openness}(S_{i})=\frac{1}{L}\sum_{j=0}^{L}r_{i,j},$$

where *L* is the length of a region (1000 bp for Deopen) and $r_{i,j}$ the number of reads that mapped to sequence region *S_i_* in the reference genome.

There are two major differences in the neural network architecture in Deopen regression model. (i) The output layer directly applies a linear transformation as $Y=W^{T}X$, since there is no discrete label available. (ii) Mean square error (MSE) is used as the loss function, since cross entropy is often used in the case of classification.

We implement the above models using the Theano framework (Bastien *et al.*, 2012) on a Linux platform. All experiments are carried out on a workstation equipped with 4 Nvidia K80 GPUs which significantly accelerated the training process compared to training on CPU.

### 2.3 Baseline models

We use two baseline models in classification. First, we download Basset, a deep learning method for predicting the genome accessibility (Kelley *et al.*, 2016), from its web site (https://github.com/davek44/Basset). Second, we download gkm-SVM (Lee *et al.*, 2015) from its web site (https://github.com/Dongwon-Lee/lsgkm). Default parameters are used for both methods.

We use three regression models (Linear, Ridge, Lasso) from the Scikit-learn library (Pedregosa *et al.*, 2011) with default parameters. Since these methods are not capable of automatically learning features from DNA sequences, we regress the openness value against manually extracted *k*-mer features, where *k* is changed from 6 to 10, and the one with the highest performance is selected.

### 2.4 Evaluating SNPs using Deopen

We apply Deopen to evaluate functional effects of genetic variants. Given a specific cell line, we train a Deopen regression model with related DNase-seq data. For a SNP, we determine a region of 1000 bp long around the SNP and predict openness values, $p^{\text{ref}}$ and $p^{\text{alt}}$, for the corresponding reference and alteration sequences, respectively. We then define a functional implication score, Δp, for the SNP as the absolute value of the difference between the two predictions, i.e. $\Delta p=|p^{\text{ref}}-p^{\text{alt}}|$.

### 2.5 Deopen motif visualization strategy

We convert a kernel of the first convolutional layer into a PWM by scanning along input sequences for activated positions of the kernel and then calculating the PWM by pooling corresponding regions. We regard a position *i* as being activated if

$$\sum_{m=0}^{M-1}\sum_{n=0}^{N-1}w_{mn}^{k}x_{i+m,n}>\alpha \cdot \text{EAV},$$

where α is the control coefficient (0<α<1) and EAV the extreme activation value defined as

$$\text{EAV}=\sum_{m=0}^{M-1}\max(w_{mn}^{k}\;|\;0\leq n\leq N-1).$$

We set length of filters in the first convolutional layer to 20 and α to 0.7 in our visualization experiments. We identify putative motifs using the tool TomTom 4.11.2 (Gupta *et al.*, 2007) with *E*-value threshold 0.05 to match PWMs identified by our method to the JASPAR database (Mathelier *et al.*, 2016).

## 3 Results

### 3.1 Deopen predicts binary accessibility status

We first designed a series of experiments to systematically evaluate the performance of Deopen in capturing genome accessibility codes from the viewpoint of binary classification. For this objective, we selected 50 cell lines at random from the ENCODE Project (Dunham, 2012), trained Deopen, Basset (Kelley *et al.*, 2016) and gkm-SVM (Lee *et al.*, 2015) on each of these cell lines, and then assessed these methods in terms of two criteria: the area under the receiver operating characteristic curve (AUC) and the area under the precision-recall curve (auPR).

**Fig. 2.**
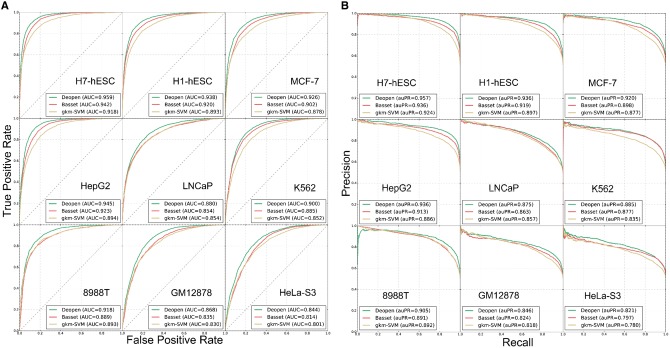
The performance of Deopen and existing state-of-the-art methods in DNA accessibility classification experiments. Deopen outperforms the state-of-the-art methods Basset (Kelley *et al.*, 2016) and gkm-SVM (Lee *et al.*, 2015) in all 50 randomly selected cell lines. We only show the results in nine typical cell lines here (See all the results in Supplementary Fig. S1). (**A**) The receiver operating characteristic (ROC) curve of three approaches. (**B**) The precision-recall (PR) curve of three approaches

According to these criteria, Deopen achieves the highest performance among all the three methods with the mean AUC of 0.906 across all the 50 cell lines, compared to 0.869 of Basset and 0.852 of gkm-SVM. The mean auPR of Deopen (0.899) also surpasses both Basset (0.863) and gkm-SVM (0.851) (see Fig. 2 and Supplementary Fig. S1). With a false-positive rate (FPR) cutoff 0.1, Deopen achieves a mean true positive (TPR) of 0.489, relative to 0.413 of Basset and 0.437 of gkm-SVM. All these results support the superiority of our method over existing state-of-the-art approaches. Besides, both a binomial exact test and a Mann–Whitney test suggest that the advantage of our method is statistically significant (Supplementary Table S1). Furthermore, considering that accessible regions account for only a small fraction of the human genome, we conducted the above comparison on unbalanced datasets (positive: negative = 1: 10) and found that our method also achieves the highest performance with an average F1-score of 0.678, relative to 0.498 of Basset (Supplementary Table S2). 

In order to evaluate contributions of CNN and *k*-mer features to Deopen, respectively, we performed a model ablation analysis where we ran Deopen in the same 50 cell lines without CNN or *k*-mer features (Supplementary Fig. S2). After removing the three-layer feed forward neural network with *k*-mer input, the mean AUC decreases about 1%. However, the mean AUC drops about 9% when removing the CNN architecture. Obviously, CNN is the most importance component in the architecture of Deopen.

To sum up, Deopen is superior to baseline methods in binary classification tasks, implying that the integration of different representations of high level features, such as *k*-mer features and those extracted by CNN, could better learn the DNA sequence code.

### 3.2 Deopen recovers continuous degree of accessibility

In the above classification experiments, we simply consider the binary status, open (accessible) and closed (inaccessible), of an input DNA sequence. However, degrees of accessibility of DNA sequences may differ from each other even when they have the same binary labels. Such difference in the degree of accessibility implicates that binary classification models are unable to discriminate putative open regions with different accessibility. To address this problem, we built a Deopen regression model to further recover the degree of accessibility of a DNA sequence. With the consideration that the accessible regions tend to contain more mapped reads. We define ‘openness’ (see formula in Methods), the degree of accessibility of a region, as the average reads mapped back to the region, thus providing a continuous measure for chromatin accessibility. We then modified the structure of our Deopen model by replacing the softmax layer with a linear transformation layer. Besides, we use mean square error (MSE) as the loss function, thus forming the Deopen regression model.

**Fig. 3.**
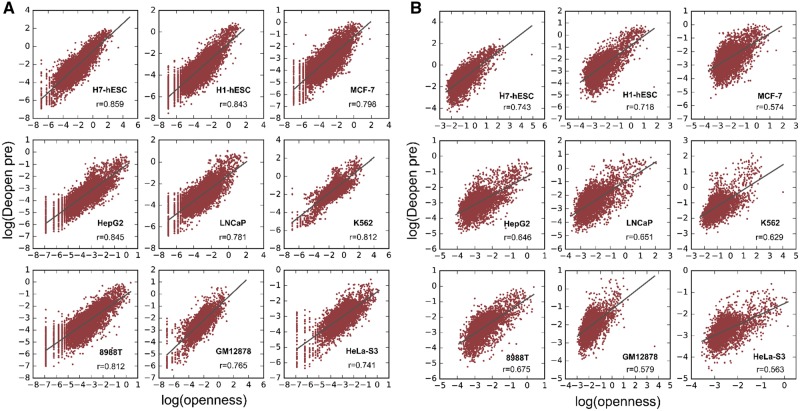
Scatter plots of regression experiments using Deopen across different cell lines. The *x*-coordinate denotes the openness we defined in Methods, and the *y*-coordinate denotes the openness our model predicted (both are in logarithmic coordinates). The Pearson Correlation Coefficient is marked in each plot. We only show the results in nine typical cell lines. (**A**) We predicted openness of both positive samples and negative samples in balanced dataset. (**B**) We predicted openness of only positive samples

Similar to the experiments in classification, we used Deopen regression model to recover the openness of input DNA sequences with the same datasets. Note that accessible regions of human genome only cover a small proportion (Kellis *et al.*, 2014). Therefore, we first predicted the openness of DNA sequences from the original test datasets, with both positive and negative samples included, across different cell lines (Fig. 3A). The obvious distribution around a straight line implicates the effectiveness of our regression model. Our Deopen regression model achieves a mean Pearson Correlation Coefficient (PCC) of 0.809 across all cell lines. The PCCs surpass 0.8 in more than half of the cell lines. 

Since people are more interested in the degree of accessibility for open regions, we removed negative samples in test datasets and directly predicted openness for the positive samples (Fig. 3B). Due to the significant difference in openness value of positive and negative samples, it is more challenging to predict the openness of positive samples alone. However, our Deopen regression model still achieves a decent outcome with a mean Pearson Correlation Coefficient of 0.648 across all cell lines. It even achieves PCC higher than 0.7 in 34% of the cell lines.

As there is no published work to predict open chromatin signals in continuous value. we compared our Deopen regression model with three regression models, Linear Regression (Galton, 1886), Ridge (Hoerl and Kennard, 1970) and Lasso (Tibshirani, 1996). We used *k*-mer feature as the input of the three baseline models. For regression with mixed samples, Deopen outperforms three baseline methods by a large margin with an average PCC 0.809 (Table 1). The small *P*-values of binomial exact test and Mann–Whitney test further support the superiority of our method (Supplementary Table S3). To verify the robustness of Deopen, we changed ratio between negative and positive samples from 1 to 10. Our method also achieves the best performance in regression experiment in all cases (Supplementary Fig. S3C). Classification models we stated before could help us judge whether the input DNA sequence is accessible. However, with Deopen regression model, we could further determine and quantify the open degree of the input DNA sequence with a continuous value. Deopen regression model hence provides us a broader way of predicting genome accessibility and inferring genome state.

**Table 1.** Deopen regression compared to other three methods

Methods	Mixed samples			Positive samples only		
	Mean	Median	Max	Mean	Median	Max
Deopen	**0.809**	**0.805**	**0.859**	**0.648**	**0.645**	**0.755**
LR	0.750	0.752	0.813	0.613	0.604	0.726
Ridge	0.753	0.755	0.813	0.616	0.604	0.727
Lasso	0.620	0.638	0.715	0.503	0.423	0.632

*Note*: Deopen regression could achieve significant higher Pearson Correlation Coefficient than other three methods in both two types of experiments. The best PCC is showed in bold. See detailed distribution of PCCs in Supplementary Figures S3A and S3B.

### 3.3 Deopen recovers known TF binding motifs

**Fig. 4.**
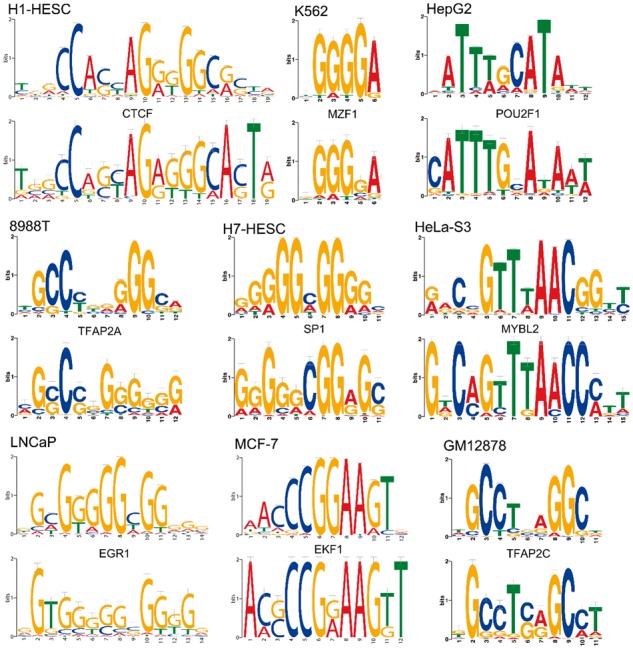
Visualization of motifs learned by Deopen from the first convolutional kernels. For each cell type, we display matched motifs with a *E*-value threshold 0.05 in the format of sequence logos (above: known motif from the JASPAR database, below: motif learned by Deopen)

To make Deopen model more interpretable and convincing, we proposed a strategy to visualize motifs learned from the first convolution layer (see Methods). We then compared these motifs with known Vertebrates motifs in the JASPAR database (Mathelier *et al.*, 2016). Using motif comparison tool TomTom (Gupta *et al.*, 2007) with significant *E*-value threshold 0.05, we match about 28 to 43% of motifs learned by Deopen in the first convolution layer to known motifs in different cell lines (see Fig. 4 for some examples). To name a few, Deopen recovers CTCF, a common architectural protein which prefers to bind in open regions (Shlyueva *et al.*, 2014), in a stem cell line (H1-HESC). In a prostate cancer cell line (LN-CaP), Deopen recovers EGR1 which is believed to be the potential target of gene therapy for prostate cancer (Baron *et al.*, 2006). In another liver cancer cell line (HepG2), Deopen recovers POU2F1 which could promote cell proliferation and inhibit apoptosis of liver cancer cells (Liu *et al.*, 2016). To sum up, the powerful learning ability of Deopen could not only help us find potential TFs binding in specific cell line, but also guide us to find novel motifs which are not discovered by experiments yet. 

### 3.4 Applications of Deopen to GWAS

To demonstrate the application of Deopen to GWAS, we collected a set of genetic variants associated with breast cancer from a previous study (Cowper-Sal *et al.*, 2012). Briefly, this dataset contains 44 SNPs associated with breast cancer, among which 29 are related to the modulation of FOXA1, a DNA-binding proteins crucial for nucleosome positioning and chromatin accessibility (Eeckhoute, 2006; Long *et al.*, 2010). Besides, there are 1057 SNPs having strong linkage disequilibrium (*r*^2^ > 0.8) with the 29 SNPs.

**Fig. 5.**
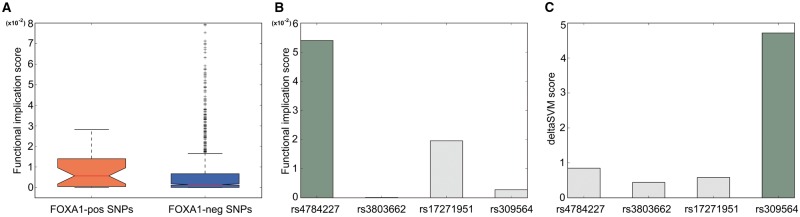
(**A**) Deopen could well discriminate 29 SNPs which modulate FOXA1 binding from the rest of 1057 SNPs that have strong linkage disequilibrium with the 29 SNPs (One-sided Mann–Whitney U test, *P*-value = 1.63 × 10^-3^, versus 0.19 in deltaSVM). (**BC**) Deopen correctly prioritizes the causal SNP rs4784227 which are believed to disrupt the binding of FOXA1 among its linked SNPs while deltaSVM failed

To show the ability of our method in discriminating the 29 SNPs against the 1057 SNPs, we identified a breast cancer cell line (MCF-7) in the ENCODE project and trained a Deopen regression model using DNase-seq data of this cell line. We then calculated functional implication scores for these SNPs and drew box plots for the 29 and 1057 SNPs, respectively. As shown in Figure 5A, scores for the 29 SNPs related with the modulation of FOXA1 are apparently higher than those of the 1057 SNPs (one-sided Mann–Whitney U test *P*-value = 1.63 × 10^−3^). In contrast, deltaSVM, a similar scoring method proposed in gkm-SVM (Lee *et al.*, 2015), yields a *P*-value of only 0.19.

Among the 29 SNPs, rs4784227 is believed to disrupt the binding of FOXA1 (Cowper-Sal *et al.*, 2012; Long *et al.*, 2010). In our dataset, there are three SNPs (rs3803662, rs17271951, rs309564) in strong linkage disequilibrium with rs4784227. According to our method, the functional implication score of rs4784227 is much higher than those of the other three SNPs (Fig. 5B). In contrast, deltaSVM is unable to correctly prioritize rs4784227 (Fig. 5C).

Furthermore, it has been shown that the risk allele, rs4784227[T], yields a 9% affinity increase when compared to the reference allele, rs4784227[C] (6.24 versus 5.73) (Cowper-Sal *et al.*, 2012). With the use of Deopen, we predict that the risk allele has a 13.8% increase of the functional implication score when compared to the reference one (0.445 versus 0.391). This result hence indicates that our method can also well predict the direction of affinity change in SNP evaluation. 

## 4 Discussion

Predicting functional elements in the genome has become a fundamental problem in computational biology. Our work has implicated that the evolution in software (CNNs), hardware (GPUs) and genomic big data have enabled drastically performance boost on such problems. Specifically, we introduced Deopen, an open source framework that integrates a deep convolutional neural network (CNN) and a feed forward neural network, to automatically learn the regulatory code of DNA sequence and implicate nucleotide driving activities. Our model has substantially surpassed the present state-of-the-art methods in the prediction accuracy. The downstream applications have already given us two scenarios with considerations of both genomics and genetics. Researchers can not only use our method to learn the chromatin accessibility code of different cell lines but also evaluate genetic variants with potential influence on the accessibility.

Besides, we have designed a series of extra experiments to verify the extensibility, scalability and robustness of Deopen. First, we further test Deopen on MNase-seq datasets which supposed to be more accurate than Dnase-seq datasets. Our method also achieves higher performance than all baseline methods (see Supplementary Fig. S4). Second, instead of using samples selected from background genome at random as the negative set, we applied two new background models (see Supplementary text S1 and S2) with considerations of GC content and cell line specificity, respectively. Deopen outperforms other methods under both new background models (see Supplementary Figs S6 and S7).

Certainly, our model can further be improved from many aspects. First, the great ability of Deopen to capture the regulatory code of DNA sequence could help us to identify other functional elements in genome, including enhancers, silencers, repressors, insulators and so forth. Second, Deopen could also be generalized to predict the impact of mutations and prioritize functional variants, thereby facilitating both research and practice of precision medicine. Third, our current model mainly focuses on the identification of accessible regions in a cell line, with the incorporation of informative data from biological experiments such as RNA-seq and ChIP-seq, it is hopeful that our model can be generalized to make cross cell-type predictions of regulatory elements. Finally, our motif analysis has showed that the first layer convolutional architecture is an effective motif discoverer. Researchers could use our method to learn the complex grammar of TFs binding in specific cell lines. It is also interesting to see whether higher layers of CNNs contain information about interactions of motifs.

To sum up, with Deopen, researcher could perform a single sequencing assay (DNase-seq, ATAC-seq, MNase-seq, etc.) of the cell type with interests. Then, one can simultaneously learn the regulatory code of genome and annotate the impact of every possible mutation in the genome. Using large-scale pubic data, one could train an accurate and interpretable model to predicting the impact of genetic variants associated with human diseases, especially the variants that lack enough interpretation in non-coding regions. We hope our approach could help unveil the regulatory mechanism underlying genetic signals and contribute to understanding the potential functions of SNPs.

## Supplementary Material

Supplementary Data
