# Supplementary material for: Chromatin accessibility prediction via a hybrid deep convolutional neural network
Source: Bioinformatics. 2017 Oct 23;34(5):732–8. doi: 10.1093/bioinformatics/btx679 (PMC6192215; doi:10.1093/bioinformatics/btx679)
Supplement: Supplementary Data [file btx679_supp.zip › btx679-suppl-data/Supplementary Materials.docx]

Supplementary materials for chromatin accessibility prediction via a hybrid deep convolutional neural network

Qiao Liu^1^, Fei Xia^2^, Qijin Yin^1^ and Rui Jiang^1, *^

^1^MOE Key Laboratory of Bioinformatics; Bioinformatics Division and Center for Synthetic & Systems Biology, TNLIST; Department of Automation, Tsinghua University, Beijing 100084, China., ^2^Department of Electrical Engineering, Stanford University, Stanford, CA 94305, USA.

*To whom correspondence should be addressed.

Contents

[Supplementary Texts 2](#_Toc493166363)

[Supplementary Figures 3](#_Toc493166364)

[Supplementary Tables 10](#_Toc493166365)

## Supplementary Texts

**Supplementary Text. S1**

We propose a strategy for selecting negative samples with similar GC content distribution as positive samples here. This background model is similar to the “random selection” one we mentioned in the main text, except that GC content of the negative set is guaranteed to be similar to the positive set in study.

To achieve this objective, we obey the following procedure. We first calculate minimum, maximum, and percentiles of GC content for a positive set under investigation. The resulting 101 values then partition GC content of the positive set into 100 intervals with equal number of data points. Next, we generate a number of regions at random (e.g., 10 times as many as positive regions) to obtain a pool of candidate negative regions. Finally, from the pool, we select regions to obtain a negative set, with the constraint that the number of data points whose GC content falls into an aforementioned interval should be equal to that of positive data points. With such strategy, negative set in each cell line has the similar GC content distribution as positive set (see Supplementary Fig. S6).

Then we perform classification experiments with different methods under 50 cell lines selected from ENCODE project at random. A comparison between our method and Basset using this background model is shown in Supplementary Fig. S7, which also clearly suggests that our method is superior to Basset, in that our method achieves higher AUC in all the 50 experiments for different cell lines. (mean AUC is 0.852 for our method and 0.808 for Basset). We also perform a one-sided Mann-Whitney U test to check whether the distribution of AUC scores of our method for the 50 cell lines has a positive shift against that of Basset, and the small *p*-value (1.71×10^-4^) also strongly supports the significance of this test and hence the superiority of our method. Besides, we observe that our method outperforms Basset in 44 out of the 50 cell lines, which yield a significant *p*-value (1.62×10^-8^) according to a one-sided Binomial exact test and further support the superiority of our method over Basset.

**Supplementary Text. S2**

We then propose another strategy for selecting negative samples from putative open regions in other cell lines. In this model, one holds the opinion that all functional regions are already known, but whether a region is active or not in a specific cell line of interest needs to be answered. Naturally, regions belonging to the cell line in study form the positive category, and those belonging to other cell lines form the negative category. Again, considering that the number of regions in all other cell lines is typically huge, a random sampling strategy can further be applied to select a small fraction of regions to form a negative set. This strategy is reasonable in the context that one likes to distinguish functional regions active in a certain cell line against all functional regions.

We implement such a background model as follows. From the ENCODE project, we have downloaded 210 DNase-seq datasets. Excluding the 50 datasets used in our previous experiments, we obtain 160 DNase-seq datasets (detailed in *Supplementary Table.xls*). Then, we collect all open regions in these datasets to form a common negative pool, with regions overlapping with any regions in the 50 DNase-seq datasets excluded. Finally, for each of the 50 DNase-seq datasets under test, we use open regions in the dataset as the positive set, and we sample at random the same number of regions from the common negative pool to form the negative set.

Then we perform classification experiments with different methods under 50 cell lines selected from ENCODE project at random. A comparison between our method and Basset using this background model is shown in Supplementary Fig. S8, which also clearly suggests that our method is superior to Basset, in that our method achieves higher AUC in all the 50 experiments for different cell lines. (mean AUC is 0.919 for our method and 0.831 for Basset). We also perform a one-sided Mann-Whitney U test to check whether the distribution of AUC scores of our method for the 50 cell lines has a positive shift against that of Basset, and the small *p*-value (1.01×10^-15^) also strongly supports the significance of this test and hence the superiority of our method. Furthermore, we observe that our method outperforms Basset in all the 50 cell lines, which yield a significant *p*-value (8.88×10^-16^) according to a one-sided Binomial exact test and further support the superiority of our method over Basset.

## Supplementary Figures


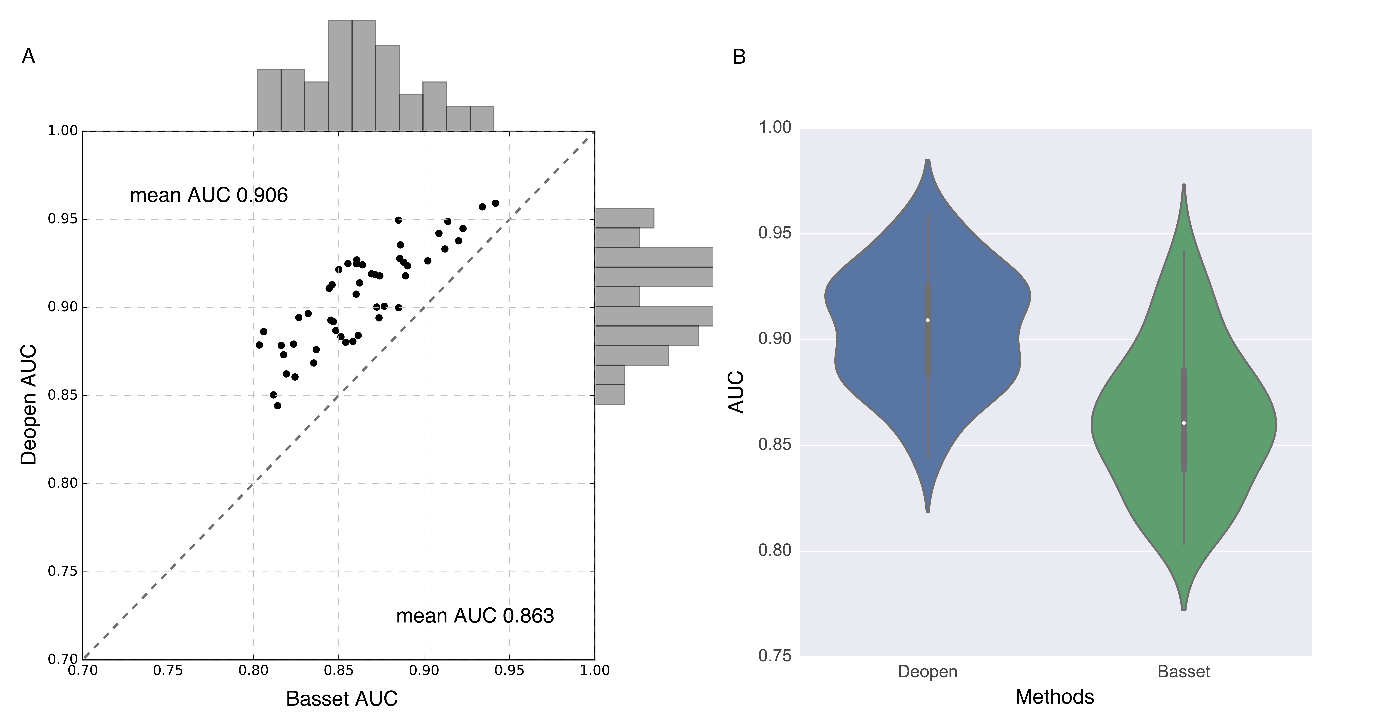


**Supplementary Fig. S1.** The performance of Deopen and Basset in 50 randomly selected cell lines from ENCODE. Negative samples are randomly selected from background genome. (A) Deopen achieves a better AUC in each cell line compared to Basset with mean AUC 0.906, relative to 0.869 of Basset. (B) Deopen could achieve a significant better performance than Basset from the AUCs distribution (Mann-Whitney U test, P-value=1.21×10^-8^).


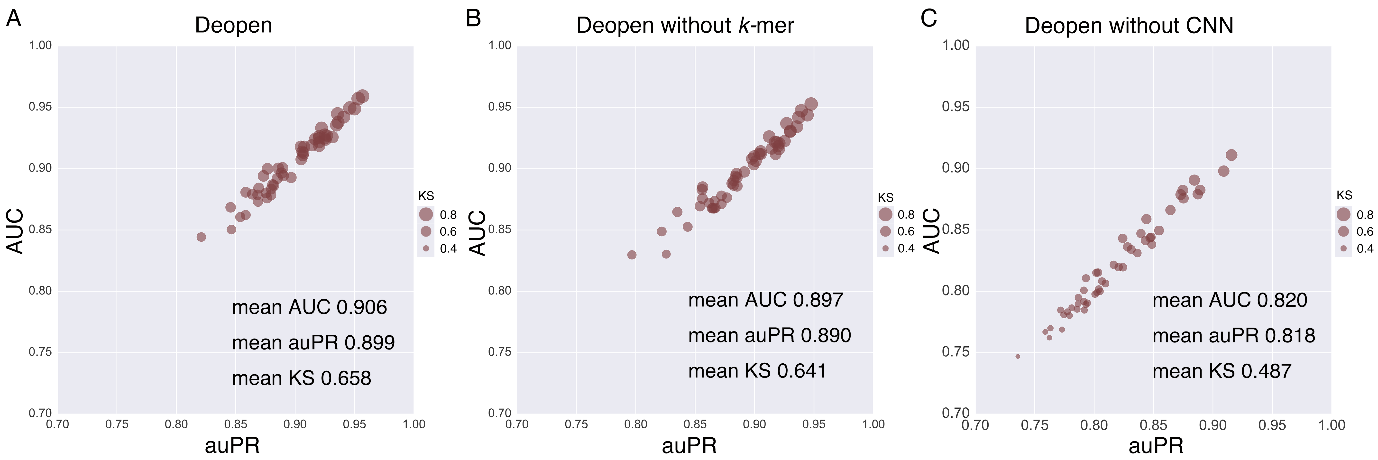


**Supplementary Fig. S2.** The contributions of CNN and *k*-mer to Deopen. We assess the contribution from the viewpoint of AUC, auPR and KS (Kolmogorov-Smirnov statistic). x-axis and y-axis denote the auPR and AUC respectively. The size of the point denotes the value of KS. After removing the three-layer BP neural network with *k*-mer input, the mean AUC and auPR decrease about 1% and mean KS decrease about 0.02. However, when the CNN architecture is discarded, the mean AUC and mean auPR decrease about 9% and 8% respectively. Mean KS decrease about 0.17. We could draw a conclusion that CNN is the most important component of Deopen.


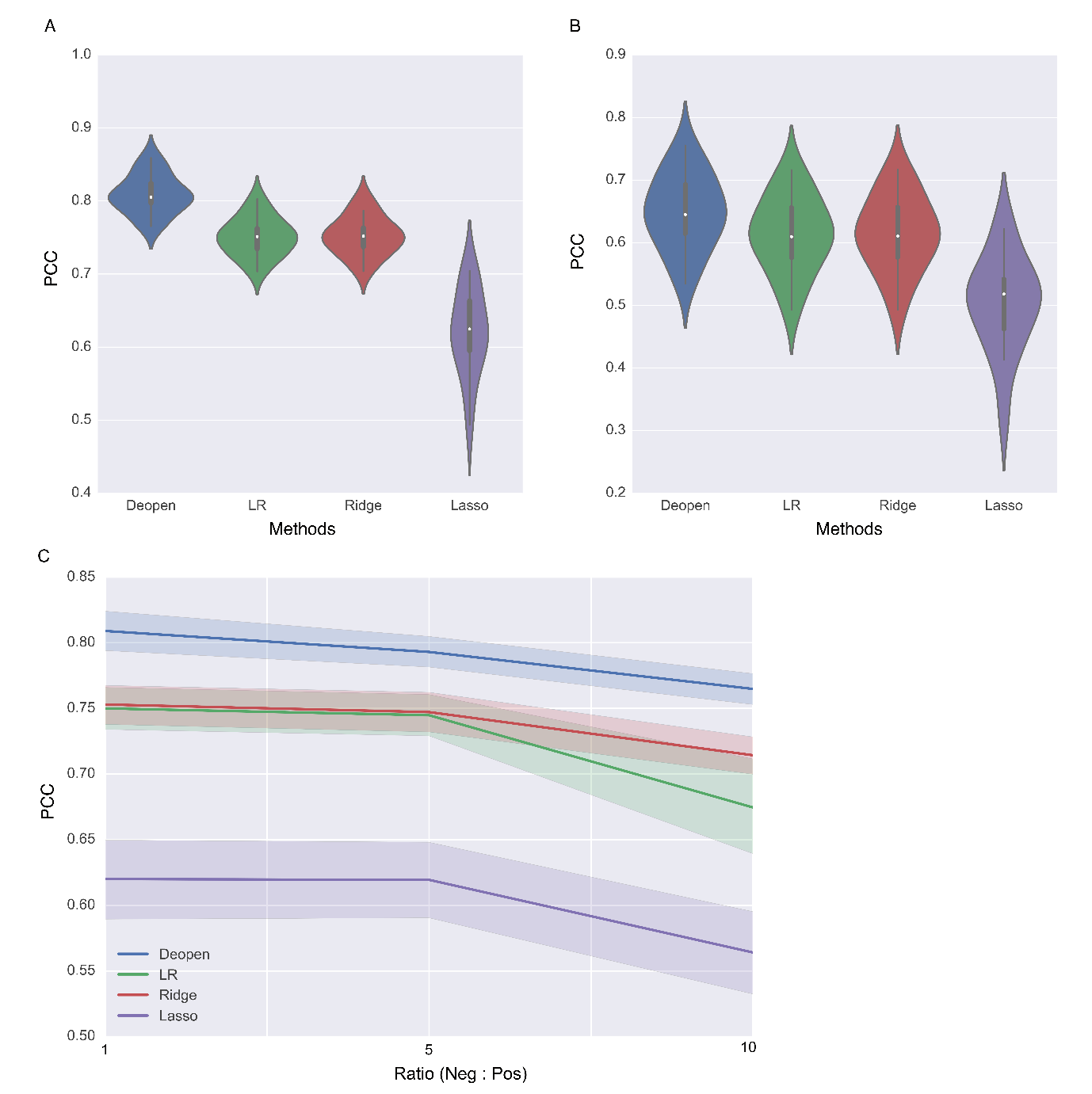


**Supplementary Fig. S3.** The distribution of Pearson Correlation Coefficient (PCC) achieved by different methods in 50 cell lines. (A) PCC distribution in balanced dataset (Mann-Whitney U test, Deopen vs LR, P-value=4.96×10^-11^). (B) PCC distribution in positive samples only (Mann-Whitney U test, Deopen vs LR, P-value=6.51×10^-3^). (C) The influence of different ratios (1,5 and 10) between negative samples and positive samples on performance of different models. Deopen and Ridge are relatively more robust compared to LR and Lasso. Deopen only drops less than 0.05 when we change the ratio from 1 to 10. The bootstrapped confidence intervals (confidence level *C*=0.9) of different methods are also shown in this figure.


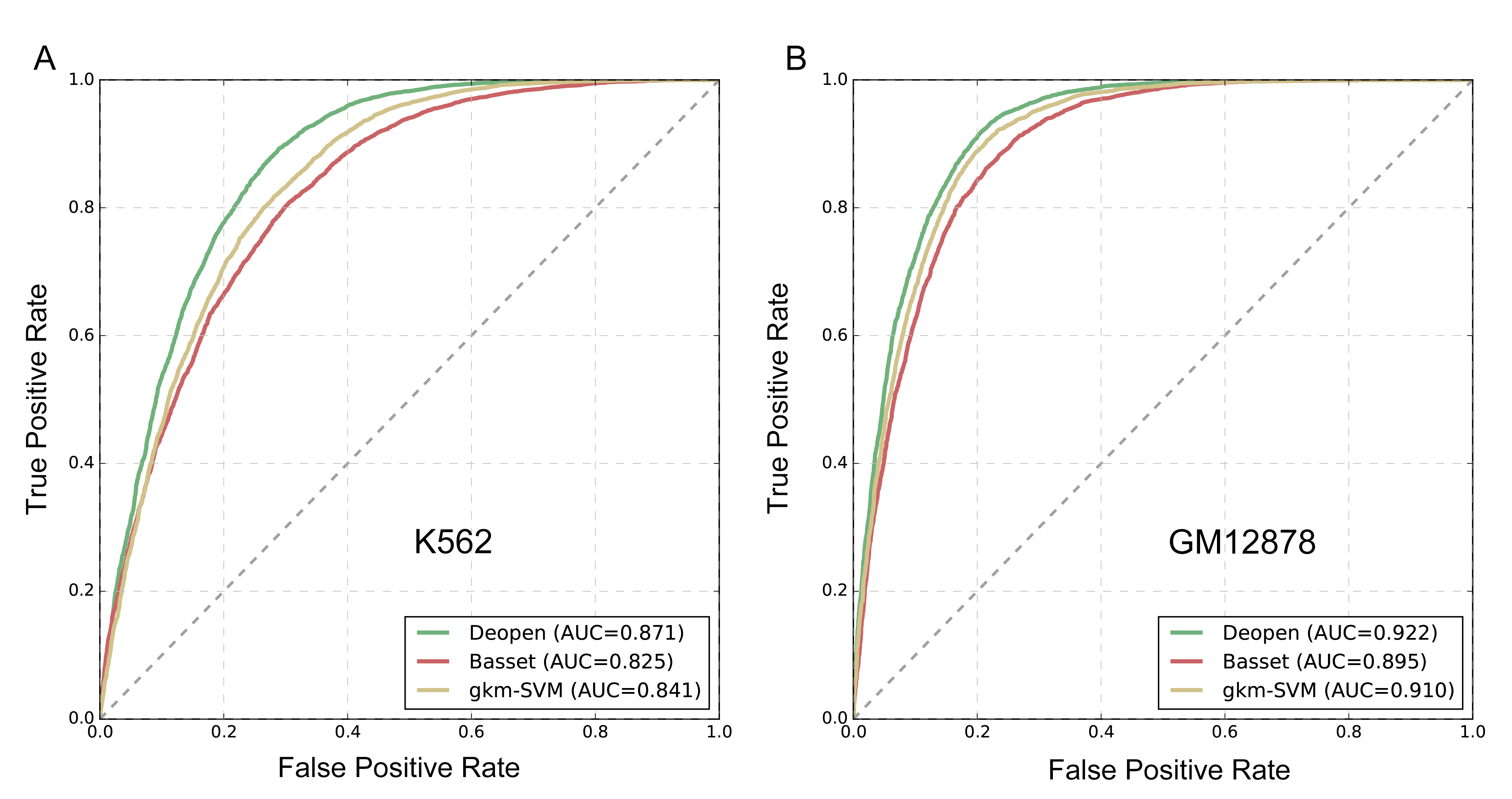


**Supplementary Fig. S4.** The classification experiments in MNase-seq datasets. We used two datasets, ENCSR000CXQ (https://www.encodeproject.org/experiments/ENCSR000CXQ) and ENCSR000CXP (https://www.encodeproject.org/experiments/ENCSR000CXP), from the ENCODE project. We preprocessed the data the same pipeline when preprocessing DNase-seq datasets. Our methods could still achieve the best performance compared to other methods.


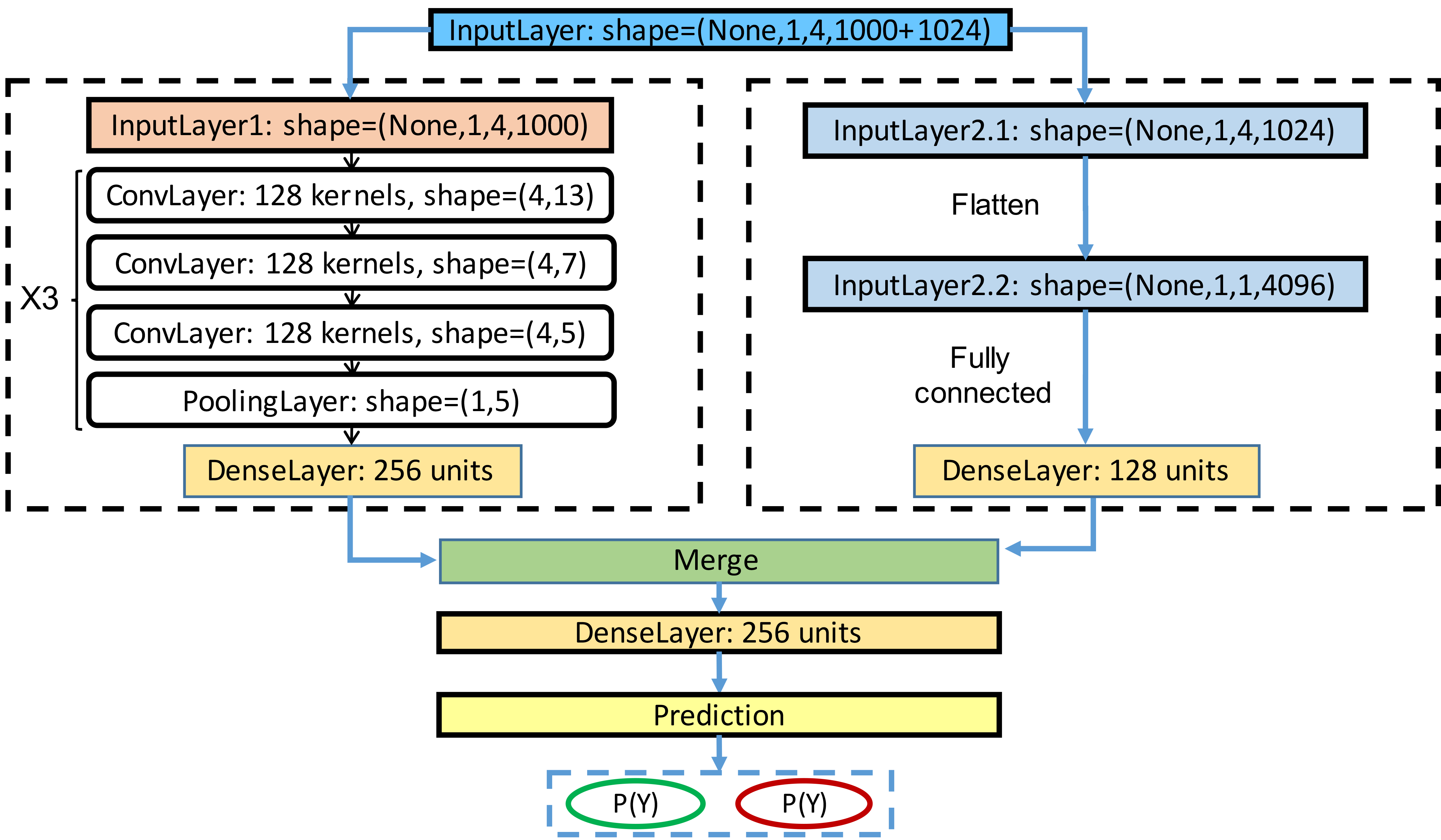


**Supplementary Fig. S5.** The architecture and model configuration of Deopen. Deopen is a bipartite model combined with CNN and a typical three-layer BP neural network. It consists of 9 convolutional layers, 3 max pooling layers, 3 fully connected layers. Each convolution layer contains 128 convolution kernels The parameter *k* is set to 6 in our model, thus creating a 1024 dimensional feature vector for each DNA sequence. We also apply dropout technology to the output of MergeLayer with the rate 0.5 in case of overfitting.


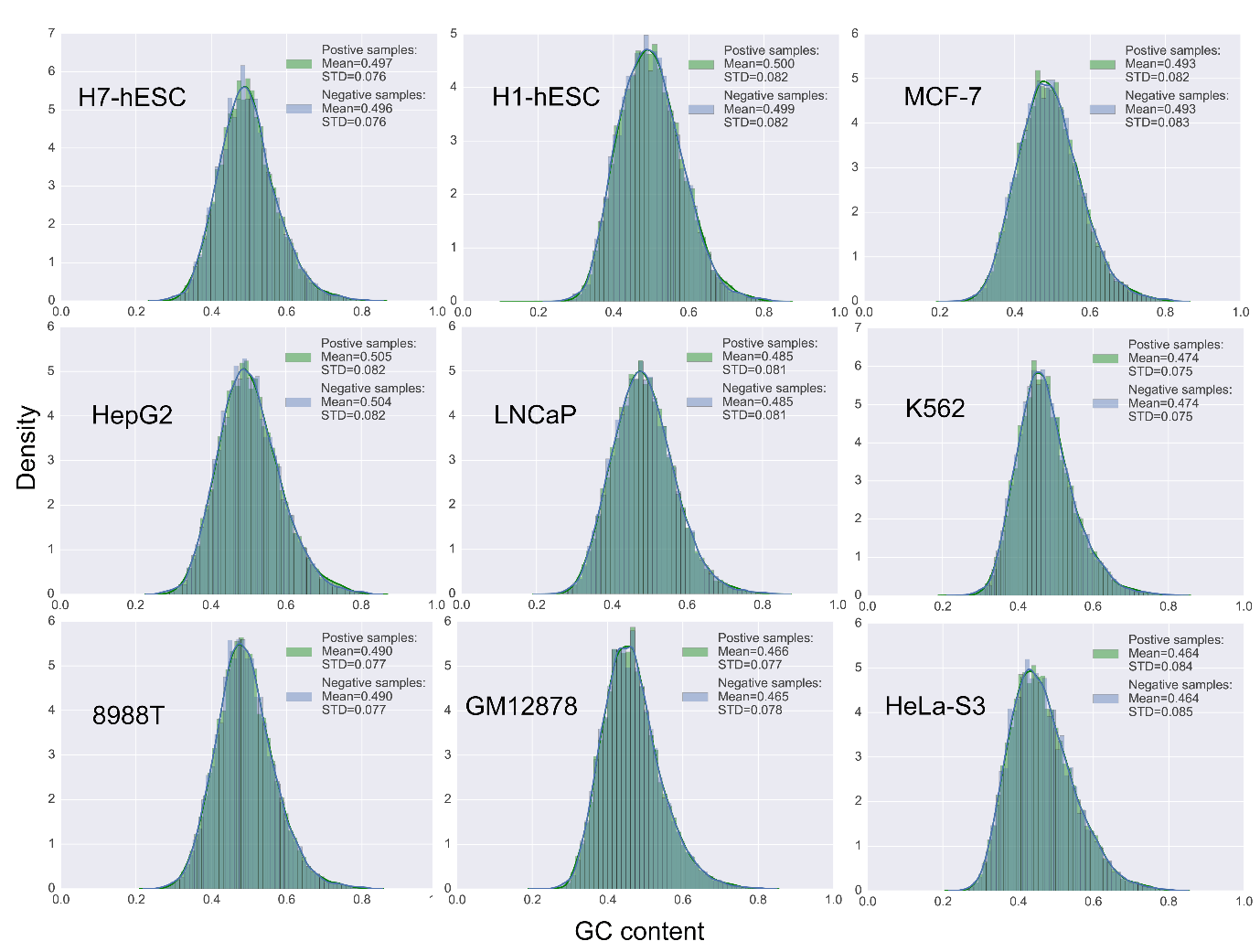


**Supplementary Fig. S6.** GC content distribution in nine typical cell lines under the random selection with GC adjustment background model. Negative set has a similar GC content distribution as positive set in each cell line.


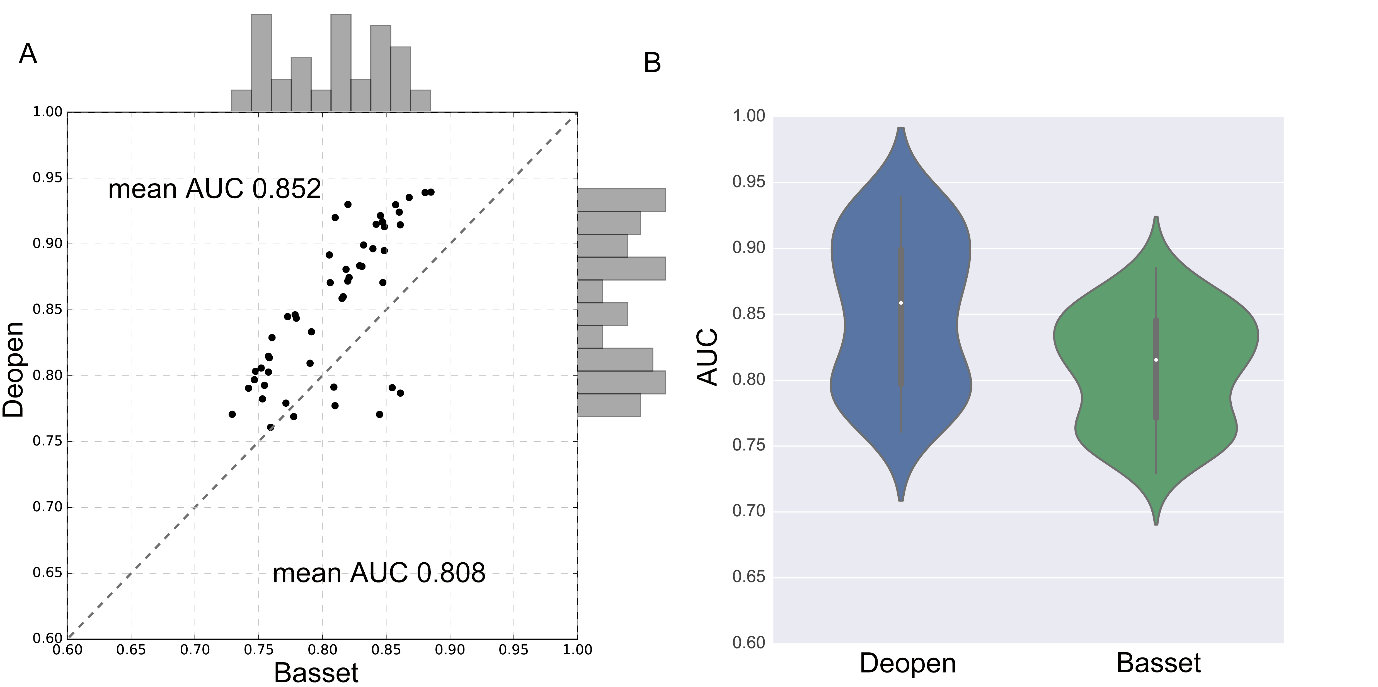


**Supplementary Fig. S7.** Performance of Deopen and Basset in 50 cell lines selected at random from the ENCODE project under the random selection with GC adjustment background model. In each cell line, negative samples have a similar GC content distribution as positive samples. (A) Comparison of AUC scores for individual cell lines. Deopen outperforms Basset in 44 out of the 50 cell lines (one-sided Binomial exact test *p*-value = 1.62×10^-8^). (B) Distribution of AUC scores. Deopen has a positive shift against Basset (one-sided Mann-Whitney U test *p*-value=1.71×10^-4^).


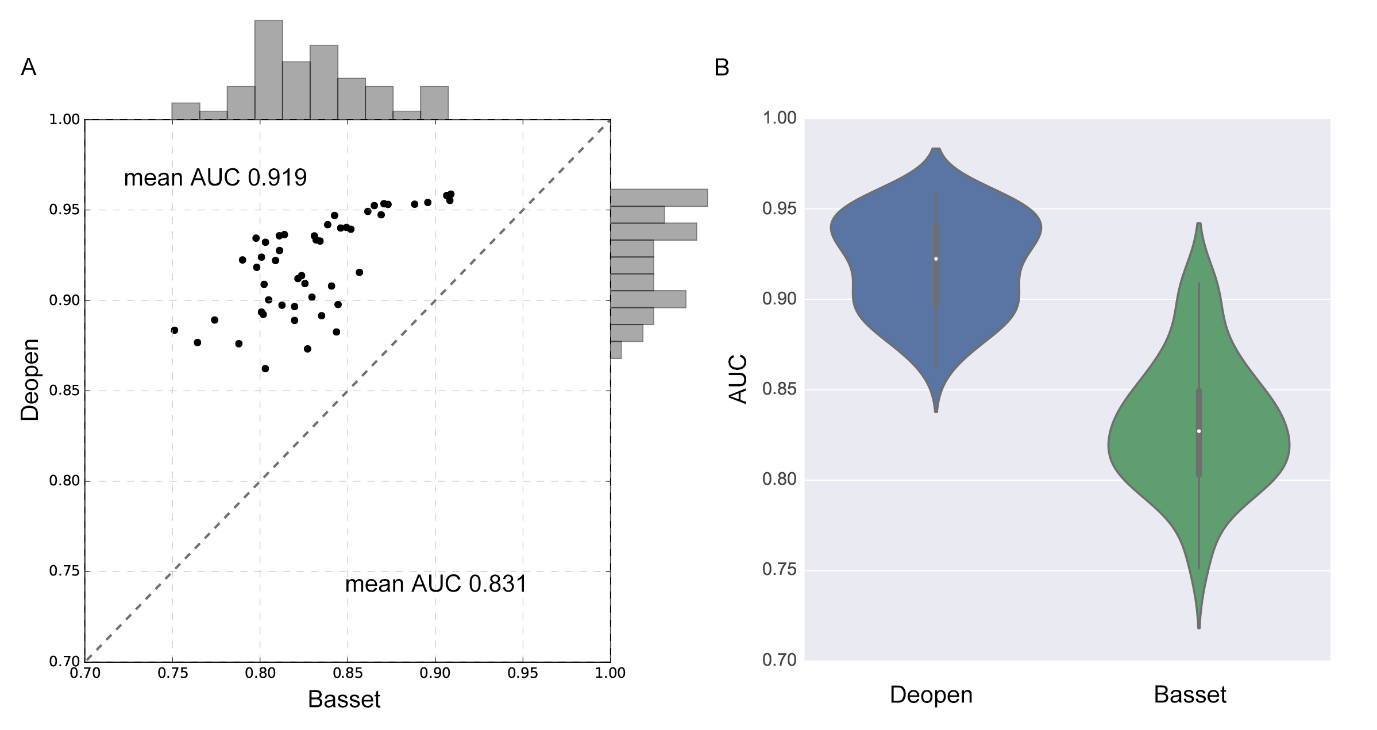


**Supplementary Fig. S8.** Performance of Deopen and Basset in 50 cell lines selected at random from the ENCODE project under the “regions from other cell lines” background model. (A) Comparison of AUC scores for individual cell lines. Deopen outperforms Basset in all the 50 cell lines (one-sided Binomial exact test *p*-value = 8.88×10^-16^). (B) Distribution of AUC scores. Deopen has a positive shift against Basset (one-sided Mann-Whitney U test *p*-value=1.01×10^-15^).

## Supplementary Tables

**Supplementary Table. S1.** P-values of two statistic tests between different methods. All the hypothesis tests are one-sided and performed based on the AUCs of 50 randomly selected cell lines from the ENCODE project. In the first test, we count the number of cell lines that our method outperforms a baseline method and then perform a binomial exact test with the alternative hypothesis that the probability that our method outperforms the baseline is greater than 0.5. In the second test, we apply a Mann-Whitney U test with the alternative hypothesis that the AUC scores of our method for the 50 cell line have a positive shift when compared with those of a baseline.

|  | Deopen vs Basset | Deopen vs gkm-SVM |
| --- | --- | --- |
| Exact binomial test | 8.88×10^-16^ | 8.88×10^-16^ |
| Mann-Whitney U test | 6.07×10^-09^ | 1.21×10^-12^ |

**Supplementary Table. S2.** Unbalanced classification metrics for Deopen and Basset models in typical nine cell lines. The F-1 score is often used for evaluating classification performance in unbalanced case. Deopen could achieve significant better performance than Basset in all unbalanced cases (Pos:Neg = 1:10).

| Metrics | Accuracy | | AUC | | F1-score | | Log-loss | |
| --- | --- | --- | --- | --- | --- | --- | --- | --- |
| Methods | Deopen | Basset | Deopen | Basset | Deopen | Basset | Deopen | Basset |
| H7-hESC | **0.961** | 0.932 | **0.971** | 0.957 | **0.785** | 0.414 | **1.356** | 2.340 |
| H1-hESC | **0.955** | 0.931 | **0.966** | 0.957 | **0.746** | 0.387 | **1.570** | 2.373 |
| MCF-7 | **0.945** | 0.940 | **0.962** | 0.958 | **0.688** | 0.595 | **1.915** | 2.064 |
| HepG2 | **0.946** | 0.926 | **0.965** | 0.948 | **0.706** | 0.633 | **1.860** | 2.546 |
| LNCaP | **0.948** | 0.923 | **0.956** | 0.935 | **0.701** | 0.378 | **1.799** | 2.647 |
| K562 | **0.932** | 0.925 | **0.946** | 0.936 | **0.634** | 0.562 | **2.339** | 2.580 |
| 8988T | **0.954** | 0.842 | **0.960** | 0.953 | **0.739** | 0.536 | **1.574** | 5.461 |
| GM12878 | **0.932** | 0.923 | **0.927** | 0.892 | **0.573** | 0.437 | **2.363** | 2.665 |
| HeLa-S3 | **0.928** | 0.907 | **0.930** | 0.880 | 0.531 | **0.537** | **2.484** | 3.228 |
| Average | **0.944** | 0.917 | **0.954** | 0.935 | **0.678** | 0.498 | **1.918** | 2.878 |

**Supplementary Table. S3.** We have applied both binomial exact test and Mann-Whitney U test to verify the superiority of our method. As for the former, we count the number of cell lines that our method achieves a larger PCC than a baseline method and then test with the alternative hypothesis that the probability that our method outperforms the baseline is greater than 0.5. As for the later, we test with the alternative hypothesis that the PCCs of our method for the 50 cell line have a positive shift when compared with those of a baseline.

**Table. S3A.** P-values of binomial exact test between different methods.

|  | Mixed samples | Positive samples only |
| --- | --- | --- |
| Deopen vs LR | 8.88×10^-16^ | 4.51×10^-05^ |
| Deopen vs Ridge | 8.88×10^-16^ | 1.52×10^-04^ |
| Deopen vs Lasso | 8.88×10^-16^ | 8.88×10^-16^ |

**Table. S3B.** P-values of Mann-Whitney U test between different methods.

|  | Mixed samples | Positive samples only |
| --- | --- | --- |
| Deopen vs LR | 2.48×10^-11^ | 3.25×10^-03^ |
| Deopen vs Ridge | 4.23×10^-13^ | 9.05×10^-03^ |
| Deopen vs Lasso | 1.53×10^-16^ | 1.45×10^-13^ |
